# Caloric restriction diminishes the pressor response to static exercise

**DOI:** 10.1186/s13728-016-0043-3

**Published:** 2016-01-20

**Authors:** John P. Florian, Friedhelm J. Baisch, Martina Heer, James A. Pawelczyk

**Affiliations:** Navy Experimental Diving Unit, 321 Bullfinch Rd., Panama City, FL 32407 USA; DLR-Institute of Aerospace Medicine, Cologne, Germany; Noll Laboratory, Department of Kinesiology, The Pennsylvania State University, University Park, PA 16802 USA

**Keywords:** Caloric restriction, Bed rest, Cold pressor, Static exercise, Sympathetic nerve activity, Spaceflight

## Abstract

**Background:**

Astronauts in space consume fewer calories and return to earth predisposed to orthostatic intolerance. The role that caloric deficit plays in the modulation of autonomic control of the cardiovascular system is unknown. Therefore, the purpose of this study was to determine the effects of 6° head-down bedrest (an analog of spaceflight) with a hypocaloric diet (25 % caloric restriction) (CR) on autonomic neural control during static handgrip (HG) and cold pressor (CP) tests. Nine healthy young men participated in a randomized crossover bedrest (BR) study, consisting of four, two-week interventions (hypocaloric ambulatory, hypocaloric bedrest, normocaloric ambulatory, and normocaloric bedrest), each separated by 5 months. Heart rate (HR), arterial pressure, and muscle sympathetic nerve activity (MSNA) were recorded before, during, and after HG (40 % of maximum voluntary contraction to fatigue), post-exercise muscle ischemia (forearm occlusion), and CP. Bedrest and nutritional combinations were compared using two-way ANOVA with repeated measures.

**Results:**

HR, MSNA, and the change in systolic blood pressure during HG were attenuated with caloric restriction, but post-intervention responses for all groups were similar during post-exercise muscle ischemia. CR was associated with a higher diastolic blood pressure during CP; however, HR was directionally opposite (i.e., increase with BR, decrease with CR).

**Conclusions:**

In summary 14-day caloric/fat restriction attenuated MSNA and pressor responses during isometric exercise to fatigue but not to post-exercise muscle ischemia. This indicates that the integrity of the metaboreflex is maintained whereas the influence of the mechanoreflex and/or central command may be reduced.

## Background

Exposure to actual [1–3] or simulated [2, 4–7] microgravity reduces tolerance for physical exertion and alters cardiovascular responses to exercise in humans. Most [2, 4–6, 8], but not all [9] studies have shown impaired reflex responses to static exercise or cold pressor tests. However, the extent to which spaceflight or bedrest influences these reflexes, or whether other factors are responsible for the modulated reflex activity, remains unclear.

By employing various stressors, it is possible to characterize afferent and efferent reflex pathways and determine how environmental adaptations (e.g., spaceflight, bedrest, fasting) modulate neural and cardiovascular responses. Static handgrip to fatigue elicits increases in blood pressure (BP), heart rate (HR), and muscle sympathetic nerve activity (MSNA) [10]. The primary mechanisms responsible for the neural and cardiovascular responses are activation of central command, a feedforward control mechanism via stimulation of the cardiovascular center from descending central neural pathways, and the exercise pressor reflex, a feedback control mechanism emanating from mechano- and metaboreceptors in skeletal muscles [10, 11]. Reflex pathways originating from cold nociceptors in the skin and involving central vasomotor centers can be assessed by sympathetic and pressure responses to the cold pressor test [9, 12].

A recent study [13] reported that astronauts were in negative energy balance (~30 %) during a 17-day shuttle mission. Hypocaloric intake reduces HR, BP, and sympathetic activity [14–16], and we have documented reduced orthostatic tolerance following caloric restriction [17]. However, the impact of reduced caloric intake alone and in conjunction with microgravity adaptation on neural control of the cardiovascular system during static exercise or cold pressor is not known. Accordingly, the purpose of this study was to test the hypothesis that caloric restriction reduces the responses to cold pressor and static handgrip exercise, and to a greater extent when combined with bedrest.

## Methods

### Subjects

Nine healthy men (age: 23.8 ± 3.0 years; BMI: 22.8 ± 3.2 kg/m^2^) completed a randomized crossover bedrest and caloric restriction study to simulate the effects of spaceflight. Approval for the project was obtained from the Ethical Committee of the ‘Arztekammer Nordrhein,’ Dusseldorf, Germany. Each subject gave written informed consent, and all procedures conformed to the Declaration of Helsinki. Subjects were enrolled if they met all of the following inclusion criteria: physical examination, ECG, urinalysis, and routine laboratory without clinically relevant findings, total cholesterol ≤200 mg/dL, LDL ≤130 mg/dL, HDL ≥35 mg/dL, and fasting glucose ≤106 mg/dL. Exclusion criteria included hyperlipidemia, arterial hypertension, diabetes, regular medication, and/or treatment with drugs within the last 6 weeks, acute or chronic illness, smoking within a period of 1 year preceding the study, and drug and/or alcohol abuse.

### Study design

**Fig. 1:**
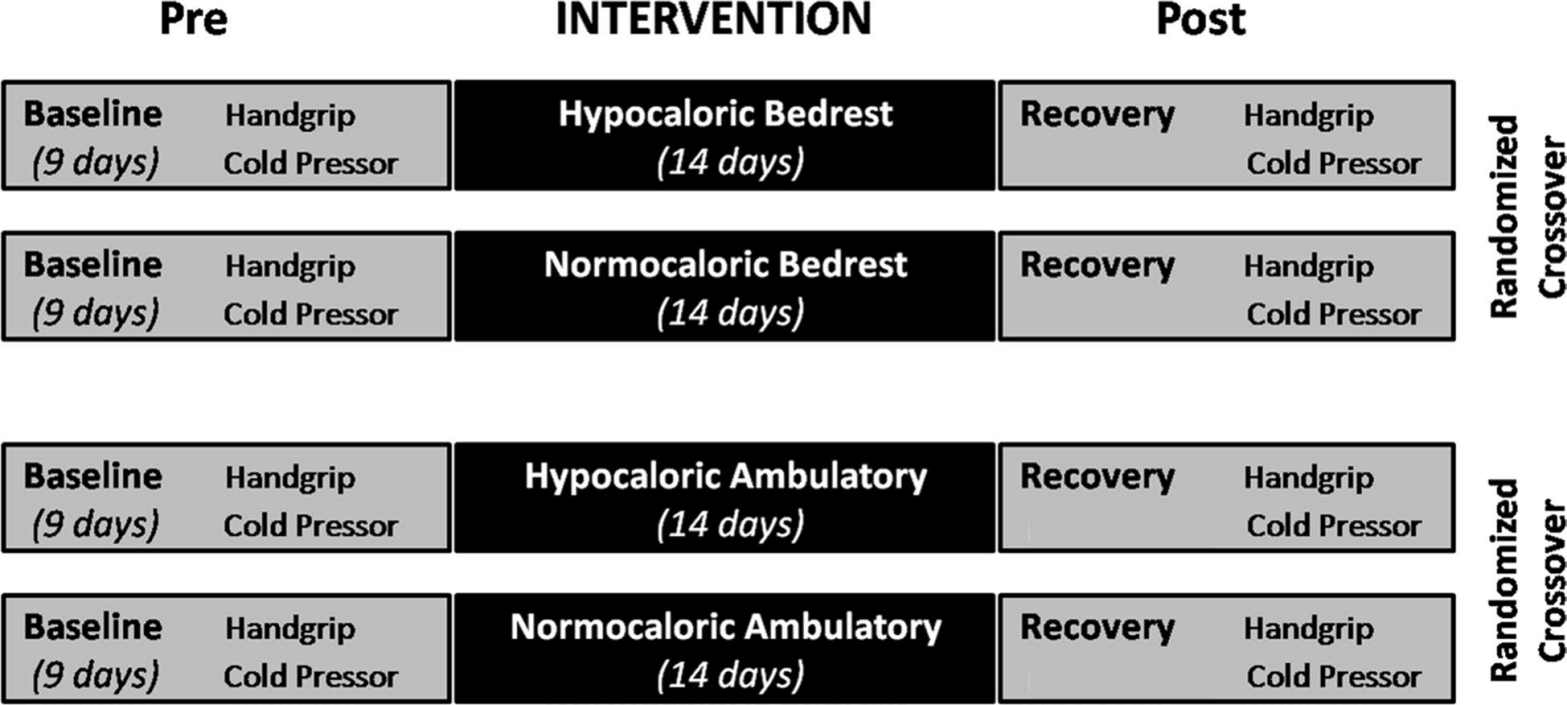
Schematic of interventions. Each subject participated in a hypocaloric and normocaloric study phase in the 6° head-down bedrest condition and in a hypocaloric and normocaloric study phase in the upright mobile condition. Each phase, which was separated by at least 5 months, started with a 9-day nutrition and physical activity adaptation period followed by a 14-day intervention period. Handgrip and cold pressor tests were completed before and immediately after the intervention

A schematic of interventions is shown in Fig. 1. The study was performed in a randomized cross-over design as part of a multi-disciplinary project evaluating the effects of simulated microgravity and hypocaloric nutrition on cardiovascular and sympathetic nervous function. Thus, information from several of the supporting references was derived from the current study design. The subjects participated in four study phases that were separated by at least 5 months to allow complete recovery of the participants, and each subject served as his own control. Each study phase started with a 9-day nutrition and physical activity adaptation period followed by a 14-day intervention period; in each of the 4 intervention periods the participants were exposed to either bedrest or ambulatory control conditions, while receiving either a tailored normocaloric or hypocaloric diet. Cardiovascular and sympathetic responses to the cold pressor test and handgrip exercise were investigated before the intervention and on day 14 of the intervention period. All four study phases were identical with respect to environmental conditions and study protocol; only the variables posture (bedrest or ambulation) and energy intake (normocaloric or hypocaloric) were changed.

### Ambulatory and bedrest conditions

The participants resided in a metabolic ward (Institute of Aerospace Medicine, German Aerospace Center, Cologne, Germany) during the entire period of the four interventions. Room temperature and relative humidity were kept constant (24 °C and 50 %) in the laboratory and metabolic ward. During the bedrest phases, all activities, including food intake, showering, using the toilet, and weighing, were carried out in the 6° head-down-tilt or horizontal position. 6° head-down-tilt was chosen because it is a validated model for simulation of microgravity [18]. Though the induced cardiovascular changes from the 6° head-down position occur more rapidly, their nature and extent are very similar to those observed in supine position. During the ambulatory control phases, the participants maintained upright position during the day and were allowed to walk around in the ward. Although they were not allowed to exercise voluntarily, they followed a light exercise protocol (including bicycle ergometry ~125 W for 15 min twice/day), a load sufficiently light to likely not have a significant impact on energy balance.

### Diet

During the 9-day adaptation periods, recovery periods, as well as during the normocaloric ambulatory intervention, the participants received a normocaloric standard diet. During the intervention periods, energy requirements were calculated for each individual according to the Food and Agriculture Organization of the United Nations and World Health Organization (FAO/WHO) equations [19] as described previously [17]. Briefly, during normocaloric conditions, participants received a diet containing 1.4 (ambulatory) or 1.1 (bedrest) times their basal metabolic rate (BMR). During hypocaloric conditions, participants received a diet containing 1.1 (ambulatory) and 0.9 (bedrest) times BMR. Ten percent of the total calories was added to account for dietary-induced thermogenesis. Dietary protein, fat (saturated and polyunsaturated fatty acids), and carbohydrate intakes were calculated according to dietary reference intake values [20] (i.e., 1.0 g/kg body mass/day as protein, 30 % as fat, and the remaining part as carbohydrates). Protein intake was kept constant during all study phases. Reduction in energy intake was mainly achieved by reduction of fat intake to a minimum level of 60 g/day in order to keep the recommended level of essential fatty acids. The remaining energy was composed of carbohydrates. Total energy and nutrient intake for all four phases has been reported previously [21].

### Heart rate and arterial pressure

Heart rate was derived from a surface electrocardiogram. Beat-to-beat finger arterial pressure was measured by finger photoplethysmography (Portapres, Amsterdam, The Netherlands), and auscultatory BP was taken at baseline.

### Muscle sympathetic nerve activity

Peroneal nerve MSNA was recorded as described previously [22]. Briefly, the nerve was located with cutaneous electrical stimulation (Isostim A320, World Precision Instruments). A tungsten reference electrode (FHC, Bowdoinham, ME, USA) was inserted subcutaneously, ~2 cm from the nerve, and a tungsten recording electrode with an uninsulated tip diameter of ~10 μm was inserted through the skin near the nerve. Adjustments of the recording electrode position were made according to auditory signals generated by impaled nerves. Both electrodes were connected in series to a differential preamplifier and an amplifier (NASA, Houston, TX, USA), isolated by two 100 mA current limiters. The nerve signal was amplified (total gain 40,000–80,000), band-pass filtered (high pass of 0.7 kHz and low pass of 2–3 kHz), and then full-wave rectified and smoothed with a resistance–capacitance circuit (time constant, 0.1 s) to produce a recording of “integrated” MSNA. Satisfactory recordings of MSNA were defined by pulse-synchronous bursts that increased during end-expiratory apnoea or Valsalva straining and did not change during tactile or auditory stimulation. Due to technical issues, MSNA was only measured and analyzed during ambulatory phases.

### Protocol

Experiments were carried out immediately before and after the 14-day intervention. Data for tests conducted following all four interventions are presented in this report. Each subject was studied while lying supine, with his lower body enclosed in a chamber made of collapsible fabric, which had zippers to access the leg for microneurography. The chamber was used for another study in the Short-Term Bedrest-Integrated Physiology (STBR-IP) autonomic investigations, and was open to air during the cold pressor and static handgrip tests. All handgrip and cold pressor tests were performed before the chamber was used to deliver lower body negative pressure. Each subject performed three brief (~3 s) maximal contractions to determine his maximal voluntary contraction (MVC) by using a handgrip dynamometer subsequent to microneurography electrode placement. The average of the three values was used as the MVC.

#### Cold pressor test

The cold pressor test was carried out after controlled-frequency breathing and Valsalva maneuvers, results of which are not included in this report. Baseline measurements were recorded for 1 min, and during the cold pressor test while the subject placed his right hand in a 0–1 °C mixture of ice and water for 2 min while maintaining a steady, relaxed breathing pattern. Immediately following the test, the subject’s hand was removed from the ice water and warmed in a towel while recovery data were recorded for 2 min.

#### Static handgrip to fatigue

After a sufficient recovery period to allow all signals to return to baseline values following the cold pressor test, baseline HR, arterial pressure, and MSNA were recorded for 1 min. Static handgrip was then performed with the dominant hand at 40 % of MVC until fatigue, followed by 2 min of post-handgrip forearm circulatory arrest with an upper arm cuff inflated to 250 mmHg, and 2 min of recovery. When the achieved force declined to <80 % of the target for ≥5 s, the cuff was inflated. During exercise, the subjects were instructed to avoid the Valsalva maneuver, as well as leg or abdominal muscle tension.

### Data analysis

Each minute of data was analyzed for the cold pressor test, and the 2 min of recovery for cold pressor and static handgrip were each averaged to a single value. Because the duration of handgrip was not constant between subjects and interventions, and since sympathetic and hemodynamic responses to static handgrip are dependent on fatigue and not actual duration, data are expressed as a percentage of total time and divided into four equal sections. Since the units for burst frequency are bursts/min, MSNA values for each stage of handgrip are normalized to 1 min.

A repeated measures analysis of variance (ANOVA) was conducted to determine the influence of caloric intake (calorie), ambulation vs bedrest (posture), and time during handgrip or cold pressor tests (stage) on MSNA and hemodynamic variables. Least squares means with Bonferroni correction were performed when appropriate to detect where differences between factors occurred. The level of significance was set at α= 0.05. Values are presented as mean ± SEM.

## Results

The subject clinical characteristics at screening are presented in Table 1. All subjects were young, healthy, normotensive, and nonobese. Subject weights, which were similar at baseline for each intervention, significantly declined following all interventions except control (hypocaloric ambulatory: 79.8 ± 3.6 vs. 78.4 ± 3.7 kg; hypocaloric bedrest: 78.7 ± 3.3 vs. 76.0 ± 3.3 kg; normocaloric bedrest: 78.1 ± 2.8 vs. 76.7 ± 2.8 kg; normocaloric ambulatory: 76.9 ± 3.2 vs. 76.7 ± 3.3 kg).

**Table 1:** Subject characteristics

Age (years)	24 ± 3
Height (cm)	182 ± 6
Weight (kg)	76 ± 7
BMI (kg/m^2^)	23 ± 9
Total cholesterol (mmol l^−1^)	4.2 ± 0.6
HDL (mmol l^−1^)	1.3 ± 0.3
LDL (mmol l^−1^)	2.5 ± 0.3
SBP (mmHg)	123 ± 6
DBP (mmHg)	78 ± 8

Values are mean ± SD

*BMI* body mass index, *HDL* high density lipoprotein, *LDL* low density lipoprotein, *SBP* systolic blood pressure, *DBP* diastolic blood pressure

### Cardiovascular response to handgrip

**Fig. 2:**
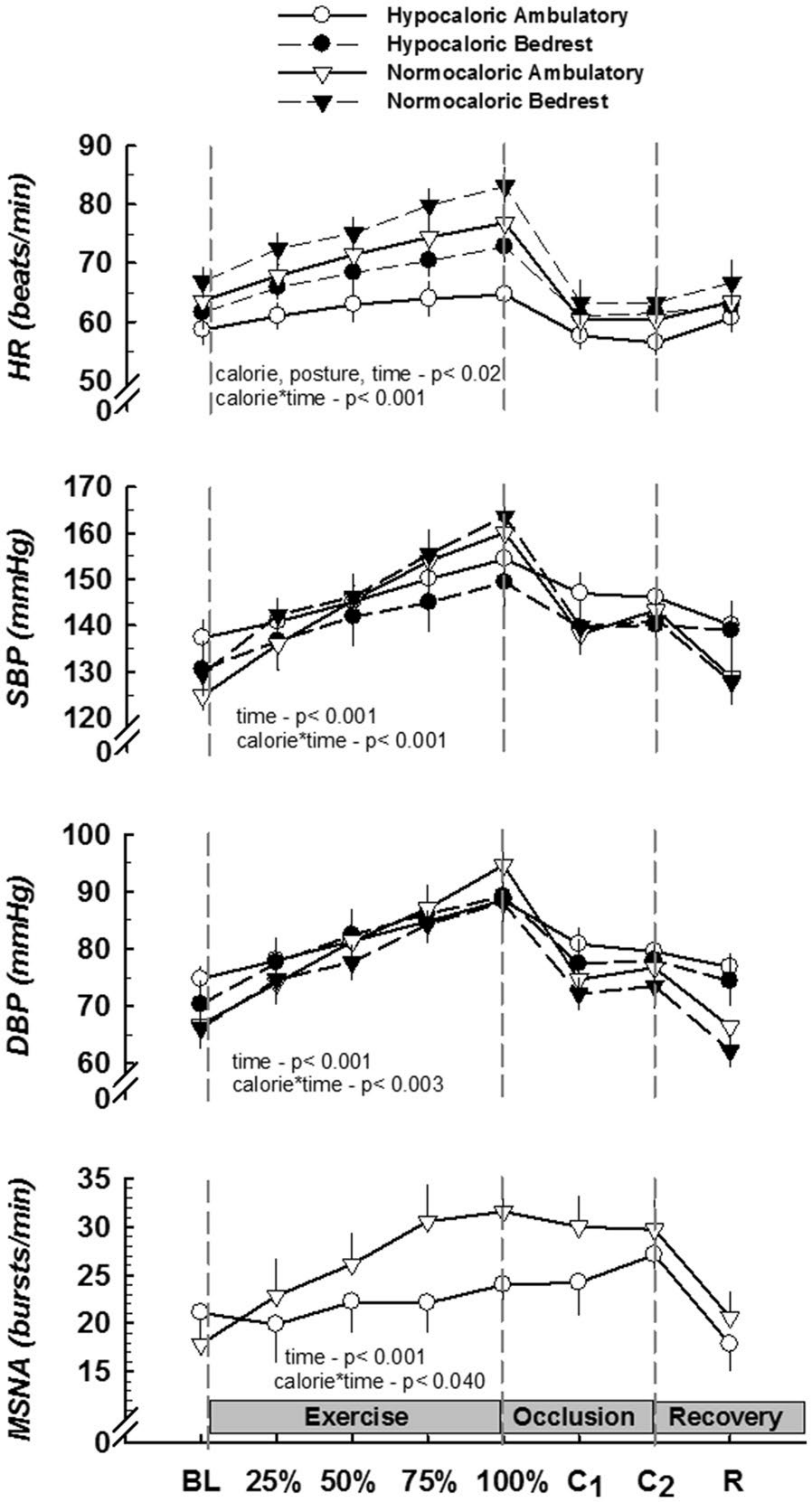
Systemic neural and hemodynamic responses to static handgrip and post-exercise muscle ischemia. Data are presented as mean ± SEM. The* x-axis* during exercise corresponds to the % of time to fatigue. *C1* and *C2*, minutes 1 and 2 of arm cuff occlusion. MSNA is adjusted to minute values and expressed as bursts/min. The main effects calorie, posture, and time are significantly different for HR. Following caloric restriction, the responses of all variables during exercise are attenuated (calorie * time interaction). Values during 2 min of occlusion are similar

**Fig. 3:**
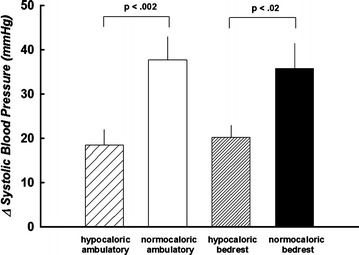
The change in SBP at the point of maximum fatigue. Data are presented as mean ± SEM. The maximum SBP response to static handgrip to fatigue was significantly attenuated following caloric restriction, independent of bedrest

The time to fatigue during static handgrip was similar following all four interventions (*p* > 0.05). Hemodynamic measurements before, during, and after handgrip and post-exercise circulatory arrest are presented in Fig. 2. Heart rate was significantly lower at baseline and throughout the protocol following caloric restriction, whereas bedrest was associated with a higher HR. At the same relative forces, HR gradually increased during static handgrip, reached its peak at fatigue, and immediately returned to baseline values during post-exercise circulatory arrest following each intervention. The contraction-induced increases in HR were diminished with caloric restriction (calorie * time interaction *p* < 0.001). Systolic and diastolic BP (SBP and DBP) increased progressively during static handgrip, peaked at fatigue, and decreased but remained elevated compared to baseline during post-handgrip circulatory arrest. The increase in DBP (Fig. 2) and SBP (Figs. 2 and 3) during handgrip were greatly attenuated with caloric restriction independent of bedrest. Responses were well maintained during post-exercise ischemia.

### Sympathetic neural response to handgrip

The MSNA responses to static handgrip and post-handgrip circulatory arrest are depicted in Fig. 2 (bottom panel). Data for only hypocaloric and normocaloric ambulatory interventions were analyzed because MSNA was not recorded during the first intervention (involving bedrest). Baseline MSNA was similar; however, the response during static exercise was significantly attenuated with caloric restriction (calorie * time interaction *p* = 0.04). Burst frequency remained elevated during post-exercise ischemia in both interventions.

### Cardiovascular and sympathetic neural responses to cold pressor

**Fig. 4:**
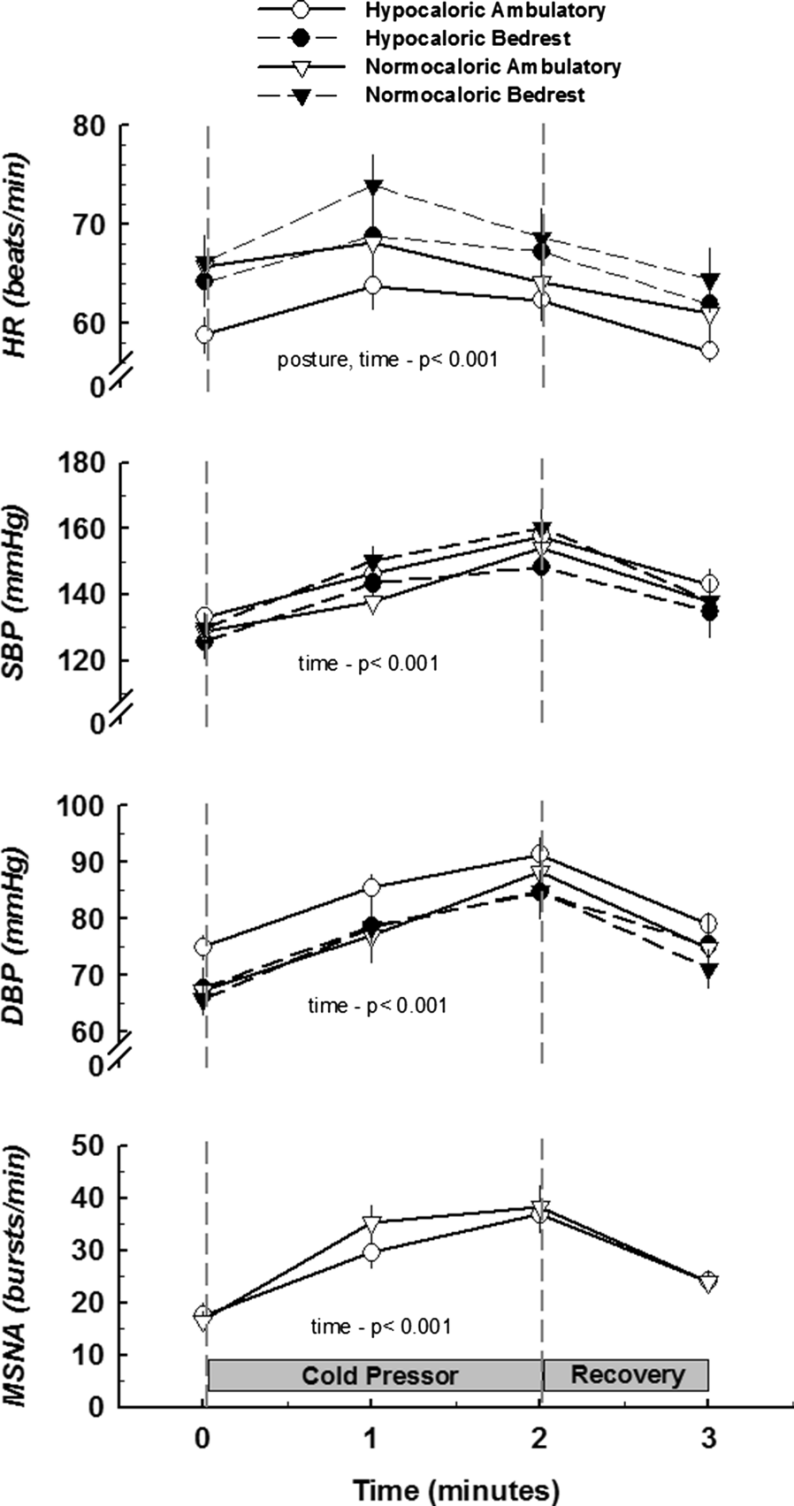
Systemic neural and hemodynamic responses to cold pressor test. Data are presented as mean ± SEM. The main effects of posture and time are significantly different for HR, whereas time is significant for BP as well as MSNA

Figure 4 shows the hemodynamic and neural responses to the cold pressor test. Heart rate at baseline and during cold pressor was increased with bedrest. As expected, the cold pressor test increased SBP, DBP, and MSNA, and levels returned to baseline following recovery. Heart rate increased during the first minute of the cold pressor test followed by a decline during the second minute and recovery. No differences in sympathetic and pressure responses were identified between any of the interventions.

## Discussion

Previous studies examining reflex neural control of the cardiovascular system following spaceflight or bedrest have reported impaired [2, 4–6, 8] or intact [9, 23] functional responses to cold pressor and/or static handgrip exercise. Therefore, the current study was conducted to determine the effects of hypocaloric intake, similar to that during spaceflight [13], on neural and cardiovascular control. We hypothesized that caloric/fat restriction alone would alter physiological responses, and that these changes would be exacerbated when caloric restriction was combined with bedrest; however, our findings support only the first part of our hypothesis.

The major findings of this investigation are fivefold: (1) HR at rest and throughout the static handgrip protocol was elevated from bedrest and reduced with caloric restriction; (2) HR and BP responses to handgrip exercise were significantly attenuated following caloric restriction trials, independent of bedrest; (3) MSNA exhibited a blunted response to exercise, but not to post-exercise circulatory arrest (metaboreceptor stimulation), following hypocaloric intake; (4) HR and BP responses after the normocaloric ambulatory and bedrest interventions were identical; and (5) HR following both bedrest trials was significantly greater at rest and throughout cold pressor; however, MSNA and BP responses were well maintained.

### Cold pressor

Although the overall HR main effect was dependent on posture, the sympathetic and cardiovascular responses to cold pressor were well maintained following caloric restriction, bedrest, and the combination of the two. This is in agreement with most [9, 23] but not all [8, 24] studies. Expected responses include a transient increase in HR within the first 30–60 s followed by sustained augmentation of BP and MSNA until termination of the test. The cold pressor test augments central sympathetic activation independent of the baroreflex and so can be utilized to test the efferent limb of the sympathetic loop [25]. Therefore, maintenance of the neural and cardiovascular responses in the current study may confirm that central reflex activation of MSNA and the corresponding vasomotor response are intact following bedrest and caloric restriction.

### Why does caloric restriction severely attenuate responses during static handgrip?

To the best of our knowledge, this is the first experiment to examine the effects of reduced caloric intake on autonomic control during isometric handgrip exercise and the cold pressor test. Our findings, though mediated by different mechanisms, are synchronous with a previous report [17] that caloric restriction reduces reflex control of the circulation during orthostatic stress. Overall, the reduced HR and BP responses are consistent with physiological adaptation to reduced caloric intake. For example, reduced caloric or fat intake lowered HR and BP in rats [15, 16, 26] and humans [24, 27]. From this investigation, it is not apparently clear why the response is drastically reduced following caloric restriction. During static exercise, activation of central command and the mechanoreflex predominantly control the increase in HR, whereas BP is regulated by mechano- and metaboreflexes together with central command, and MSNA is mainly regulated by the metaboreflex [11]. Alterations can occur at a number of points along the muscle mechano- and metaboreflex arcs (e.g., afferent response, central integration, efferent signal) and central command in addition to changes in stimuli and end-organ responses. From our results, it seems most likely that central command and/or the mechanoreflex are attenuated.

#### Central command

Immediately at the onset of exercise, central command modulates the level of sympathetic and parasympathetic efferent activity to the vasculature and heart [11]. The magnitude of control is largely influenced by the individual’s perceived effort during actual or attempted exercise, independent of absolute workload or force production. For example, increasing or decreasing central command at a given muscle tension during static exercise results in a corresponding increase or decrease in cardiovascular responses [28]. Although the exact location of integration of these signals is unknown, it appears to include regions of the insular and anterior cingulate cortexes that interact with thalamic and brainstem structures of cardiovascular integration [29]. Nutrient signaling within the hypothalamus and dorsal vagal complex that controls appetite and sympathetic outflow may modulate the influence of central command through shared neural pathways [30–32]. The blunted neural and cardiovascular responses during handgrip in this study are consistent with, but do not prove, a reduction in central command output. More mechanistic studies are needed to determine whether and how caloric restriction modulates central command.

#### Muscle mechanoreflex

The mechanoreflex, which mainly consists of group III and some group IV mechanosensitive afferents that respond to stimuli such as stretch, contraction, and pressure [33], increases HR primarily through vagal inhibition [34] and may also augment sympathetic activation [35, 36]. That the HR and MSNA responses during static handgrip exercise were reduced whereas MSNA continued to increase comparable to the normocaloric intervention during muscle ischemia collectively suggest that the mechanoreflex may be impaired.

Several possibilities may account for the impaired reflex. First, the sensitivity of muscle afferents is directly proportional to interstitial fluid [35, 37]. Therefore, a reduction in plasma volume and interstitial fluid that may occur with caloric restriction [27], bedrest [38, 39], or water immersion [40], may desensitize the mechanoreceptors. However, this seems unlikely since the cardiovascular responses to handgrip following the normocaloric bedrest intervention were similar to control. Second, caloric restriction may modulate central integration of the mechanoreflex, similar to that of central command. Third, reduced caloric/fat intake may decrease adrenergic sensitivity [24] while increasing endothelium-dependent and –independent vasodilation [41]. This may be consistent with the attenuated BP response; however, it would not explain the diminished sympathetic outflow compared to normocaloric conditions.

#### Muscle metaboreflex

Stimulation of the metaboreceptors situated in the interstitial space of muscle elicits increases in MSNA and arterial pressure [11]. The metaboreflex (as well as mechanoreflex and central command) is activated during static handgrip due to buildup of metabolites from mechanical occlusion of blood vessels by the contracting muscle; however, the reflex can be isolated during post-exercise circulatory arrest when mechanical stimulation and central command influences are absent. Since the neural and hemodynamic responses during muscle occlusion were similar following all four interventions, caloric modulation of the metaboreflex is unlikely to contribute to the reduced exercise responses.

## Limitations

Several limitations may be associated with the present study. First, microneurography was not attempted during the first phase (bedrest), limiting the MSNA analysis. Second, sympathetic outflow included only efferent outflow to skeletal muscle, so these findings may not represent sympathetic outflow to other vascular beds. Third, cardiac output and thus systemic vascular resistance were not assessed, limiting our ability to interpret the hemodynamic results. Finally, instead of randomizing the order of stressors, a serialized order, employed in previously published studies [9, 42], was chosen to minimize the impact of any order effect on pre- and post-intervention comparisons. To minimize carryover effects, the schedule allowed sufficient recovery from the cold pressor to handgrip tests. The fact that baseline and recovery data were not different from one stressor to the next suggests that this strategy was appropriate.

## Conclusion

In summary, 14-day caloric/fat restriction attenuated MSNA and pressor responses during isometric exercise to fatigue but not to post-exercise muscle ischemia. This indicates that the integrity of the metaboreflex is maintained whereas the influence of the mechanoreflex and/or central command may be reduced. Similar increases in MSNA, HR, and BP during the cold pressor test were recorded following each intervention indicating that central reflex activation may remain intact. We conclude that hypocaloric low-fat intake reduces the pressor response to static exercise. Further research will be required to (1) more clearly elucidate which portion(s) of the reflexes are impaired following caloric restriction and (2) determine the underlying metabolic/hormonal signals associated with the observed neural and cardiovascular changes.

## Abbreviations

ANOVA: analysis of variance
BMI: body mass index
BMR: basal metabolic rate
BP: blood pressure
CP: cold pressor
DBP: diastolic blood pressure
HDL: high density lipoprotein
HG: handgrip
HR: heart rate
LDL: low density lipoprotein
MSNA: muscle sympathetic nerve activity
MVC: maximal voluntary contraction
SBP: systolic blood pressure
STBR-IP: short-term bedrest-integrative physiology
